# Motor unit action potential conduction velocity estimated from surface electromyographic signals using image processing techniques

**DOI:** 10.1186/s12938-015-0079-4

**Published:** 2015-09-17

**Authors:** Fabiano Araujo Soares, João Luiz Azevedo Carvalho, Cristiano Jacques Miosso, Marcelino Monteiro de Andrade, Adson Ferreira da Rocha

**Affiliations:** Department of Electrical Engineering, University of Brasília, Campus Darcy Ribeiro, Caixa Postal 4386, 70910-900 Brasília, DF Brazil; UnB Gama Faculty, University of Brasília, Area Especial de Indústria, Projeção A, Setor Leste, Gama, 72444-240 Brasília, DF Brazil

**Keywords:** Surface electromyography, Conduction velocity, Image processing

## Abstract

In surface electromyography (surface EMG, or S-EMG), conduction velocity (CV) refers to the velocity at which the motor unit action potentials (MUAPs) propagate along the muscle fibers, during contractions. The CV is related to the type and diameter of the muscle fibers, ion concentration, pH, and firing rate of the motor units (MUs). The CV can be used in the evaluation of contractile properties of MUs, and of muscle fatigue. The most popular methods for CV estimation are those based on maximum likelihood estimation (MLE). This work proposes an algorithm for estimating CV from S-EMG signals, using digital image processing techniques. The proposed approach is demonstrated and evaluated, using both simulated and experimentally-acquired multichannel S-EMG signals. We show that the proposed algorithm is as precise and accurate as the MLE method in typical conditions of noise and CV. The proposed method is not susceptible to errors associated with MUAP propagation direction or inadequate initialization parameters, which are common with the MLE algorithm. Image processing -based approaches may be useful in S-EMG analysis to extract different physiological parameters from multichannel S-EMG signals. Other new methods based on image processing could also be developed to help solving other tasks in EMG analysis, such as estimation of the CV for individual MUs, localization and tracking of innervation zones, and study of MU recruitment strategies.

## Background

In surface electromyography (surface EMG, or S-EMG), conduction velocity (CV) refers to the velocity at which the motor unit action potentials (MUAPs) propagate along the muscle fibers, during contractions. MUAPs propagate in the direction that causes the membrane voltage of muscle fiber cells to approach and surpass the threshold for excitation, causing the action potential to move [1]. This propagation occurs along the direction of the muscle fibers, and originates from the innervation zone (IZ) in opposing directions, towards the tendinous regions. The CV is related to the type and diameter of the muscle fibers, ion concentration, pH, and firing rate of the motor units (MUs) [1–5]. The CV can be used in the evaluation of contractile properties of MUs [6], and of muscle fatigue [7–9]. Typical values of CV are in the 3 to 5 m/s range, with an average of approximately 4 m/s [1]. In clinical applications, CV can be used to supplement the information at the muscle’s fiber level obtained with needle EMG [10]. For example, van der Hoven studied CV in patients with amyotrophic lateral sclerosis using needle and S-EMG and discovered that these patients presents increased CV and decreased median frequency [11].

The CV can be estimated from S-EMG signals measured using an array of electrodes, placed over the target muscle, and parallel to the muscle fibers. Signal conditioning is typically performed using four or more electrodes in a double differential configuration. This setting is used because it provides greater reduction of end-of-fiber components, when compared to the differential configuration [12]. However, the differential configuration may also be used to estimate the CV, especially when the detection region is far from IZs and tendon regions [13].

A current approach generally used to estimate the CV is based on computing the ratio between the interelectrode distance (IED) and the delay between the S-EMG waveforms associated with two or more adjacent electrodes [12, 14–23]. The most popular methods for CV estimation are those based on maximum likelihood estimation (MLE) in the frequency domain, which provide higher velocity resolution and lower variance than other approaches [12, 23]. Most of the CV estimation methods available in the literature provide only a mean CV value [14–17, 20, 21, 23]. However, methods for estimating the CV associated with a single MU have also been proposed [12, 23]. In 2004, Farina *et al.* compared the MLE with these approaches and other common methods, and showed that MLE is the most accurate among them [23]. To the best of our knowledge, MLE is still the most used CV estimation method.

Two-dimensional (2D) features of multichannel S-EMG—where the two dimensions correspond to the electrode-array axis and the time axis, respectively—can be exploited to estimate the mean CV. Grönlund et al. has used 2D techniques to estimate the CV of a single MU, as well as muscle fiber orientation, using an optimal trajectory method [24]. The techniques in [25] are more precise and more usable in practice, but have some limitations such as poorer performance in case of MUAP superposition and requirement of *a priori* knowledge of the direction of propagation.

This paper proposes a 2D method for estimating the mean of the CV from electrode-array S-EMG signals, using digital image processing techniques. The proposed method is based on the processing of images constructed from the set of signals acquired with an array of electrodes, placed parallel to the muscle fiber. The analysis of such images allows the estimation of the delay between similar waveforms on adjacent S-EMG channels. Then, just as in the traditional methods for CV assessment, the CV is estimated as the ratio between IED and MUAP delay.

## Methods

### Multichannel S-EMG signal displayed as an image

Multi-channel electromyography is a technique for recording S-EMG signals on several skin positions over a muscle, using an array or a matrix of uniformly-spaced electrodes. When the vector configuration is used (i.e., a one-dimensional array of electrodes), the measured signals are stored as a matrix, in which the rows correspond to different channels, and the columns correspond to different time instants (or vice-versa). Such matrix may be displayed as an image, as follows.

**Fig. 1:**
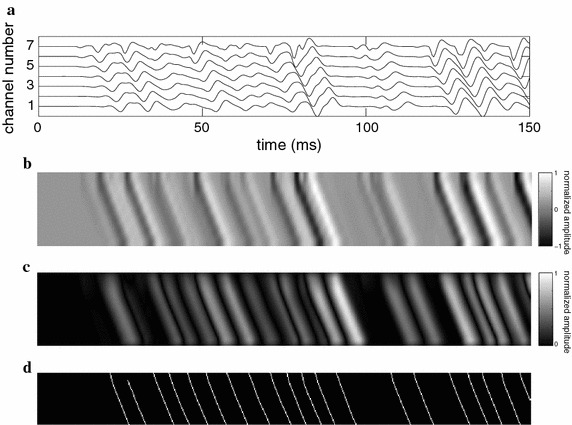
Steps of the proposed CV estimation algorithm. **a** 150-ms segment of a 7-channel synthetic S-EMG signal (without IZ). The horizontal axis represents time, and the vertical axis represents both channel number (i.e., spatial position along the electrode array) and signal amplitudes (scaled to the $[-1,+1]$ range). **b** Image representation of the multichannel S-EMG signal shown in (**a**). Bi-cubic interpolation was applied along the temporal (two-fold) and spatial (100-fold) axes. The aspect ratio was changed for display purposes only. **c** Hermite-filtered image. Note that the conduction lines are softer and have been highlighted from the background. **d** Segmented (and cropped) image. The slope of each of the segmented lines provides the CV of one MUAP. The mean CV may then be calculated for the segment

Acquisitions using a (*M*+1)-electrode array in single-differential mode will result in *M*-channel S-EMG signals (Fig. 1a). A *N*-sample segment of such a signal will correspond to a $M \times N$ matrix. Such a matrix may be displayed as a $M \times N$ image, by mapping the signal amplitude values onto a gray-scale color map. In such image, the vertical axis corresponds to the spatial position along the electrode array, the horizontal axis corresponds to time, and the gray levels represent the intensity of the measured electric potential (signal amplitude).

However, the number of channels in an array is generally not higher than 15. In this case, the image would be extremely narrow and difficult to visualize and process. This can be addressed by interpolating the multi-channel S-EMG signal along the channel axis (or along both axes), thus obtaining a matrix of a larger size, which may be more clearly visualized in image form (Fig. 1b). The interpolation in both dimensions also improves the performance of the image processing algorithm proposed in this work, by thickening the diagonal lines associated with conduction of the MUAPs, as discussed below. Generally, white and light gray pixels in the resulting images represent large positive amplitude values, while black and dark gray pixels represent large negative amplitude values; small amplitude values are represented by intermediary shades of gray.

The interpolated data, when displayed as a gray-scale image, allows the identification of the MUAPs, which form diagonal lines diverging from the rows associated with IZs. These lines represent the conduction of the MUAPs along the muscle fibers. The shapes of these conduction lines are affected by many morphological parameters of the corresponding MU, such as: (1) length and width of the MUAP line, which is associated with the depth of the MU that originated the MUAP and the number of fibers of the MU that originated the MUAP; (2) the velocity in which the MUAP travels along the MU, i.e. the CV, which is the slope of the conduction lines; and (3) the location of IZs, which may be detected as pairs of conduction lines with opposite slope, originating from horizontal gray-colored regions of the image [26]. This work, however, focuses solely on estimating the CV from the S-EMG images.

**Fig. 2:**
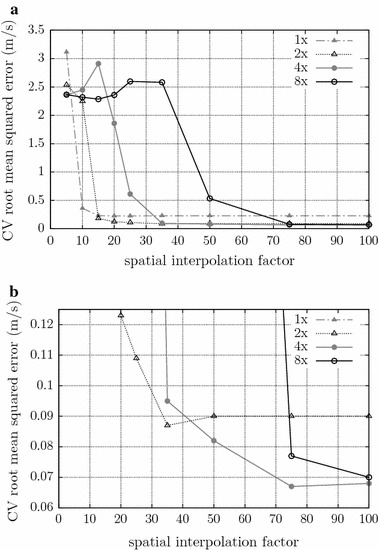
Estimation of the root mean squared error for the IPE algorithm using 100 synthetic signals with force level of 20 % of the MVC and 16 dB SNR. **a** Comparisson of the IPE performance using 1, 2, 4, and 8-fold time interpolation, and 5, 10, 15, 20, 25, 35, 50, 75, and 100-fold spatial interpolation. **b** Detail on the IPE performance evaluated in (**a**). It is possible to see that the best interpolation factor is 4-fold time interpolation, and 75-fold spatial interpolation

The S-EMG image associated with each multichannel S-EMG signal (or with each segment of one) was constructed by resizing the 2D data using four-fold interpolation along the temporal axis, and 75-fold interpolation along the spatial (channel number) axis. These interpolation factors were chosen empirically and are the best pair for minimizing error, as can be seen in Fig. 2, this figure was created using 100 signals generated by the method described on section *CV experiments using simulated S-EMG signals*. Bicubic interpolation was used, as it generally does a better job of preserving fine detail than the bilinear approach [27].

### MUAP-enhancement filter

The images obtained from the multichannel S-EMG signal show several diagonal lines of varying intensity, associated with the MUAPs (Fig. 1b). Low-intensity conduction lines are generally associated with deep MUs, or with MUs of smaller size (with few muscle fibers). Conduction lines generally do not have clear boundaries, and low intensity MUAPs may be lost if a binarization process is applied directly. In order to address this issue, a waveform-detection filter was used to intensify regions where MUAPs are present, by highlighting the edges of the conduction lines (Fig. 1c). We chose an Hermite filter for this task—after also experimenting with Gaussian and Gabor filters—, as it allowed a better delineation of the MUAPs, for the signals we tested. This approach was first introduced by us, for both CV estimation [28, 29], and IZ detection [26, 30]. More recently, Ullah et al. used Hermite filtering for MUAP-enhancement in multi-scale S-EMG processing [31].

Let *x* be the horizontal axis of the S-EMG image (time), and *y* be its vertical axis (spatial position along the electrode array). The kernel of the Hermite filter is the first derivative of a Gaussian with respect to the *x*-axis:

1 $$\begin{aligned} G_{2,1}(x,y)= -\frac{x}{2 \pi \sigma _{x}^{3} \sigma _{y}} \exp \left( -{\frac{x^{2}}{\sigma _{x}^{2}}}-\frac{y^{2}}{\sigma _{y}^{2}}\right) , \end{aligned}$$

where $\sigma _{x}$ and $\sigma _{y}$ are the standard deviations (in number of pixels) about the *x* and *y* axes, respectively, and define the kernel width [32].

This filter will emphasize MUAP conduction lines that are above or below an IZ, without the need to consider the direction of propagation of the MUAPs. We used $\sigma _{x} = \sigma _{y} =$ 60 pixels; these values are consistent with the typical duration of MUAPs [33] and proposed interpolation factors. With this configuration, the Hermite filter is able to highlight the differences between regions of inactivity and regions where MUAPs are present (Fig. 1c). These values were chosen based on a compromise between the size of the filter, and edge effects created by the filter response (ripple artifacts). We found this setting to be the most suitable for all the experiments we conducted. The proposed values can be changed according to the needs of a particular experiment or application. To validate the filter tuning, we estimate CV of 100 simulated S-EMG signals, the simulation process is described in the section *CV experiments using simulated S-EMG signals*. In this validation process $\sigma _{x}$ and $\sigma _{y}$ values were changes from 10 to 100 in steps of 5 independently, and the value of 60 for both parameters was the one that provides most precise results.

### Conduction velocity estimation

The proposed image processing estimation (IPE) algorithm for CV estimation is based on calculating the slope of the MUAP conduction lines on segmented S-EMG images. The algorithm consists of the following steps:

- The S-EMG image is constructed from a multichannel S-EMG signal (or a segment of one), using the previously described process (Fig. 1b).
- An Hermite filter is used to improve the contrast of the MUAP conduction lines (Fig. 1c).
- The image is binarized. This eliminates background noise, and allows the use of binary morphological operations. The threshold is set to the median amplitude value of the Hermite-filtered image.
- A morphological opening operation is performed. This operation aims to erase noisy (irregular and grainy) lines, typically associated with problematic regions of the image (e.g., motion artifacts).
- A morphological thinning operation is performed, in order to reduce the width of the MUAP conduction lines. Morphological thinning removes pixels from an object, until a line where all pixels are connected is obtained. This results in diagonal lines that can be used to estimate the CV (Fig. 1d).
- All objects shaped like the letter “H” are broken, in order to remove connections between two conduction lines.
- Isolated pixels are removed.
- The image is cropped (edges are removed), in order to avoid distortions due to filtering and interpolation (e.g., Gibbs artifacts).
- Conduction lines are automatically labeled by an object detection algorithm, which finds and labels each image element that is not connected to other elements.
- The slope of each conduction line is estimated, using least squares linear regression. This is the measured CV for the associated MUAP.
- All MUAPs with mean squared error (MSE) (with respect to the corresponding regression line in the channel direction) greater than 0.6 mm$^2$ are eliminated. This is because lines with MSE larger than this threshold are usually associated with false, noise-related objects.
- Lines shorter than the value of the inter-electrode distance (5 mm) are discarded.
- Vertical lines (slope greater than 20 m/s) are discarded. These lines do not represent MUAP propagation, and therefore must not be considered. (typical CV values ranges from 3 to 5 m/s).
- The mean CV is calculated as the weighted average of the CV values of all conduction lines which were not discarded. The weight of a conduction line is directly proportional to the square of its length (total number of pixels in the line)—i.e., smaller weight is attributed to conduction lines that were detected by few electrodes—, and inversely proportional to the sum of the variances of the *x*-axis and *y*-axis regression errors, respectively—i.e., smaller weight is attributed to conduction lines that are composed by pixels that deviate significantly from a line.

Note that classification algorithms could be used (prior to binarization) to identify different MUs, and thereby estimate the CVs of individual MUs. The shapes, intensity, and position of each MUAP could be used for this classification. We propose this idea as a potential future work.

### Evaluation of the proposed algorithm

The performance of the proposed IPE algorithm was quantitatively and qualitatively evaluated by comparison with the MLE algorithm proposed by Farina et al. [12]. This algorithm estimates the CV by finding the waveform delay between adjacent channels that minimizes the MSE between the channels’ waveforms and a template signal, which is modeled as the mean of the waveforms measured on all channels, after delay compensation. It is important to mention that the initial CV value for the MLE algorithm was set to 4 m/s, and that we assumed that the propagation direction was known. IPE, however, does not require a choice of initial CV value, nor *a priori* knowledge about propagation direction; in fact, IPE estimates the CV for both directions of propagation, and does not require any kind of initialization.

#### CV experiments using simulated S-EMG signals

A total of 750 synthetic signals were generated, using the simulation method proposed by Farina and Merletti [34]. Each simulated signal contains seven differential channels. The signals were designed based on a hypothetical muscle with 200 mm length, fat layer of 6 mm, skin layer with 1 mm, and 30 motor units, each with a random number of muscle fibers (ranging from 50 to 550 fibers) and MU recruitment threshold of 100 % of the MVC. All signals were simulated far from tendinous regions and innervation zones, and based on a single narrow IZ, so that the simulated MUs are innervated at the same location. The simulated IZ was positioned far from the electrodes. The sampling rate was 2048 Hz (per channel). The simulation was based on a set of 8 linear electrodes contacts with 5 mm IED. The total duration of each signal was 3 s.

These signals were separated into eighteen groups of 50 synthetic signals, which were used to compare the two algorithms. Signals from each group had different CV values (3, 4, or 5 m/s), null standard deviation, different signal-to-noise ratio (SNR) values: $\infty$ (noise free), 30, 20, 16, or 12 dB and different force levels (20, 40 and 60 % of the MVC). The CV for each signal from each group was estimated using both MLE and IPE algorithms. The results were then quantitatively and qualitatively compared between algorithms, and with the true CV for each signal, by means of scatter plots and root mean squared error (RMSE) levels.

The S-EMG signal may be segmented into several windows prior to CV estimation. This may be particularly useful in studies on muscle fatigue, in which the temporal behavior of the S-EMG estimators (including CV) during an exercise is evaluated [35]. The CV for a window is estimated as the average of the CVs of the individual MUAPs within that window. Reducing the window length improves the algorithm’s temporal resolution. However, such reduction may have the drawback of reducing the algorithm’s precision and/or accuracy, because fewer CV measurements would be averaged in each window, i.e. the sample size would be reduced. The influence of the window length on the algorithms’ precision was evaluated using the same simulated signals used in the previous experiment, but performing the CV estimation process multiple times, each time with a different window length. The results were evaluated by comparing RMSE levels. Just 4 m/s noise free and 16 dB SNR signals were used for this analysis. The window length used were 0.5, 0.75, 1.0, 1.5, or 3 s, the RMSE was calculated from the CV values estimated in all windows—6, 4, 3, 2, or 1 window, respectively—from all 50 signals in the set.

We also analyzed the influence of the number of channels used to estimate the CV for both algorithms. The number of channels was varied between 4 and 7. In this experiment, the CV was 4 m/s, and the window length was 3 s, for all signals. This evaluation was performed at two different noise levels: noise-free and 16 dB SNR. The results were evaluated by comparing RMSE levels.

When analyzing the results of these CV experiments using simulated S-EMG signals, we will consider that the CV estimates are accurate and precise if (and only if) the RMSE is lower than 0.1 m/s. Such low RMSE allows evaluating statistically significant differences between the CV estimates of different MUs, thus allowing the estimation of the CV of a single MU [12]. Therefore, we define our “high-goodness threshold” at 0.1 m/s. If the RMSE is higher than 0.1 m/s, but lower than 0.5 m/s, than it may not be possible to distinguish different MUs, but it is still possible to detect variations of the average CV [12]. Thus, we define our “low-goodness threshold” at 0.5 m/s; i.e., we will consider that the CV estimates are inaccurate and/or imprecise if (and only if) the RMSE is greater than 0.5 m/s.

#### CV experiments using biceps brachii signals

Finally, both algorithms (IPE and MLE) were used to estimate the CV in biceps brachii S-EMG signals. Twenty-three females and eighteen males volunteered to participate in the study. Due to withdrawal, hormonal problems, failing channels, fail in sustain isometric strength level and high levels of noise in the S-EMG recordings, only sixteen female volunteers (24.1 ± 2.5 years old) and eleven male volunteers (25.8 $\pm$ 2.6 years of age) were included in the analysis. All subjects were right-handed and had no known neurological disease. All female subjects had regular menstrual cycles, did not practice regular exercise, and were not using any medication or hormonal contraception for at least 6 months. Male volunteers also did not practice regular exercise. All volunteers read and signed an informed consent form. The experimental protocol was approved by the research ethics committee of the University of Brasília.

**Fig. 3:**
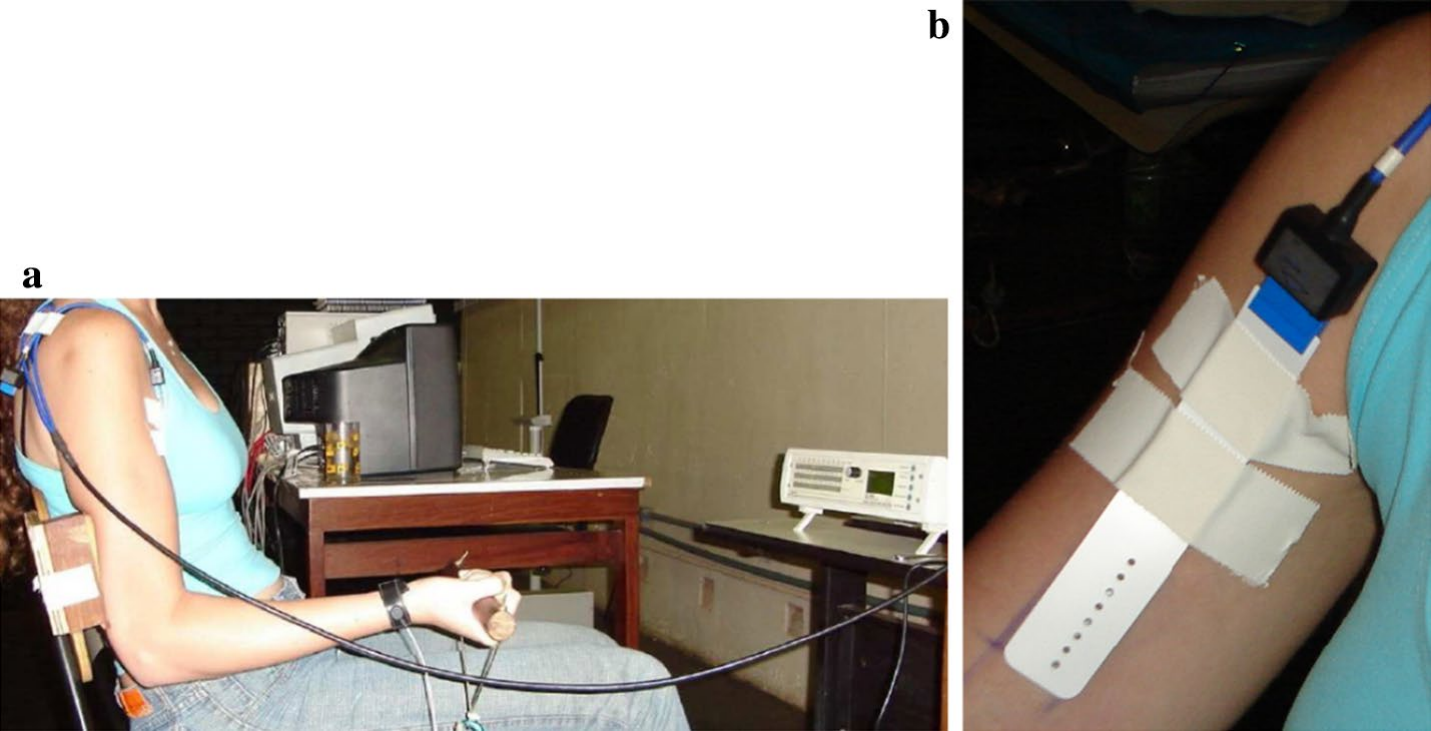
Experimental setup. **a** Position of the subject during exercise. A chair was adapted so that the elbow joint was flexed at an angle of 90°, and to secure the elbow so that the only possible movement of the arm was isometric elbow flexion. The load cell was attached to an adjustable cable, and placed next to the chair. **b** Placement of the 7-channel electrode array, on the ideal region of acquisition of the biceps brachii short head, after innervation zone identification

The same experimental protocol was executed with all the volunteers. Each female subject performed the experimental protocol in four sessions, with a one week interval between sessions. Male volunteers underwent a single session. Each volunteer sat on a chair, specially adapted to secure the elbow, so that the only possible movement of the arm was isometric elbow flexion (Fig. 3a). This was done to minimize muscle contractions that could impact the results of the experiment.

In order to acquire the EMG signals, we used the EMG 16 electromyograph by OT Bioelettronica snc, Italy, connected to a laptop through a PCMCIA card. The EMG 16 uses a 4th-order bandpass Bessel filter with passband between 10 and 500 Hz, and provides a maximum sampling frequency of 2048 Hz. A model-TS 50-kgf load cell (Aeph Brazil, São Paulo, Brazil), connected to a MISO II biomechanical signal amplifier (OT Bioelettronica snc, Italy), was used to measure the strength level of the subjects. The amplification gain was set to 1.

Three 3-second isometric contractions were performed at the beginning of each session to determine the volunteer’s MVC. However, for female volunteers, the MVC measured during the first session was adopted as the MVC for all sessions; MVC values of other acquisitions were measured only to ensure the repeatability of the protocol in all sessions. Strong verbal encouragement was used for each MVC. After each MVC estimation, the subject rested for 1 min.

Then, in each session, an array of 16 electrodes (Ag, size: 10 $\times$ 1 mm, 5 mm inter-electrode distance, OT Bioelettronica snc, Italy) was used to map the ideal region of the biceps brachii muscle for the acquisition of the S-EMG signals [36]. For this assessment, the sampling rate was 2048 Hz per channel, the gain was set to 1000, an analog differential configuration was used, and the strength level was 30 % of the MVC. Three-second signal acquisitions were used for mapping the ideal position. After this mapping procedure, the volunteer rested for two minutes.

After mapping, S-EMG signals were acquired with a semi-disposable linear array of eight surface electrodes (5 mm inter-electrode distance, OT Bioelettronica snc, Italy), which was placed on the optimal region (between IZ and tendon regions) of the biceps brachii short head (Fig. 3b). The skin was cleaned and conductive gel was applied between the skin and each electrode. The differential configuration was used, resulting in a 7-channel S-EMG signal for each acquisition. A reference electrode was placed on the right wrist. The sampling rate was 2048 Hz, and an the analog gain was set to 1000. A 10-second acquisition using 20 % of the MVC was performed. The subjects rests than for 2  min and finally, a 90-s acquisition using 40 % of the MVC was performed.

A total of 27 signals were selected from those measured during the 10-s contractions (signals from the IZ mapping procedure were not used for CV estimation tests). Signals that presented poor quality in at least one channel—60 Hz interference, movement artifacts, loss of electrode contact (intermittent or constant)—were discarded. The signals were not segmented (i.e., 10-s window length), and seven channels were used for obtaining the S-EMG images. In these experiments, the true CV is unknown. Thus, CV estimates obtained with the proposed method were compared with those obtained with the MLE algorithm, by means of correlation coefficient, linear regression, and scatter plots analysis.

## Results

### CV experiments using simulated S-EMG signals

**Fig. 4:**
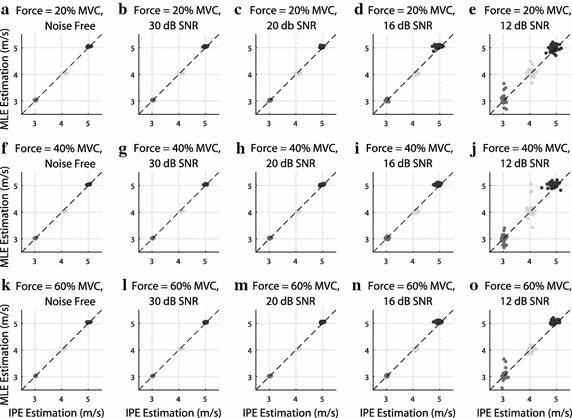
CV estimation comparison between the proposed IPE algorithm and the MLE algorithm proposed by Farina et al. [12], using simulated signals with different CV values—3 m/s (*medium gray*), 4 m/s (*light gray*), and 5 m/s (*dark gray*)—, different levels of noise: **a** noise free; **b** 30 dB SNR; **c** 20 dB SNR; **d** 16 dB SNR; and **e** 12 dB SNR, and different level of force: 20 % MVC, 40 % MVC and 60 % MVC. The *black diagonal dashed lines* are the identity *lines*. Each MLE–IPE pair of CV measurements (one dot) is associated with one simulated signal

Figure 4 and Tables 1 and 2 show the results of the CV estimation comparison between the proposed IPE algorithm and the MLE algorithm proposed by Farina et al. [12], using simulated signals with different levels of noise, different CV values and different force levels.

**Table 1:** RMSE of IPE algorithm for synthetic signals with CV equal to 3, 4, and 5 m/s, force level equal to 20, 40 and 60 % of the MVC and SNR of $\infty$ (noise free), 30, 20, 16 and 12 dB

CV (m/s)	$\infty$ dB SNR	30 dB SNR	20 dB SNR	16 dB SNR	12 dB SNR
Force level = 20 % MVC					
3	0.02	0.03	0.04	0.04	0.05
4	0.09	0.09	0.10	0.10	0.10
5	0.03	0.03	0.04	0.09	0.14
Force level = 40 % MVC					
3	0.03	0.03	0.04	0.05	0.05
4	0.09	0.09	0.09	0.09	0.08
5	0.03	0.03	0.04	0.08	0.17
Force level = 60 % MVC					
3	0.03	0.03	0.04	0.04	0.05
4	0.08	0.09	0.09	0.10	0.09
5	0.03	0.03	0.04	0.07	0.12

**Table 2:** RMSE of MLE algorithm for synthetic signals with CV equal to 3, 4, and 5 m/s, force level equal to 20, 40 and 60 % of the MVC and SNR of $\infty$ (noise free), 30, 20, 16 and 12 dB

CV (m/s)	$\infty$ dB SNR	30 dB SNR	20 dB SNR	16 dB SNR	12 dB SNR
Force level = 20% MVC					
3	0.03	0.03	0.04	0.04	0.15
4	0.04	0.04	0.04	0.04	0.12
5	0.05	0.05	0.05	0.06	0.15
Force level = 40% MVC					
3	0.03	0.03	0.03	0.04	0.13
4	0.04	0.04	0.04	0.05	0.24
5	0.04	0.04	0.04	0.06	0.08
Force level = 60% MVC					
3	0.03	0.03	0.03	0.04	0.16
4	0.04	0.04	0.04	0.04	0.08
5	0.04	0.05	0.05	0.06	0.09

In Fig. 4, we show MLE versus IPE scatter plots for each noise level, with groups with different nominal CV values shown in different colors in each scatter plot. The black diagonal dashed lines represent the identity line; therefore, if the dots are grouped near the diagonal line, this indicates that the two algorithms provided similar CV estimates.

Tables 1 and 2 present the same results in a different fashion, by evaluating the effects of the noise level and force level on the RMSE for each algorithm, and for each CV value.

These results show that, when assessing signals with high SNR ($\ge$16 dB), both methods were able to estimate the CV for all signals with high accuracy and precision (RMSE $\le$ 0.1 m/s) i.e. the results in this SNR range are within the high-goodness threshold defined in the methodology.

At low SNR ($\le$12 dB), the MLE method presented results within the low-goodness threshold, defined in the methodology (0.08 m/s $\le$ RMSE(MLE) $\le$ 0.24 m/s, with RMSE(MLE) the root mean squared error of the MLE). In some MLE estimations it is possible to see the spreading of the estimated CV value in Fig. 4e, j, o. This is due to local minima problems associated with the Newton–Raphson method, which is used in the MLE algorithm. The proposed IPE method does not use iterative optimization methods, therefore it is not susceptible to this type of error. The IPE method has a slightly better performance for 12 dB SNR (0.05 m/s $\le$ RMSE(IPE) $\le$ 0.17 m/s, with RMSE(IPE) the root mean squared error of the IPE). However, there is no spreading behavior in the IPE case, as opposed to the MLE results.

Another important aspect we observed is the fact that the applied force level seems to have little effect on the performance of both the IPE and the MLE methods, specially for high SNRs. In fact, Tables 1 and 2 show that for signals with 30 and 20 dB, there is no difference between the measured root mean squared errors for different force levels, within the considered numeric precision. In the case of signals with 12 dB, there were different RMSE (for the MLE only) for different tested force levels, but the error sometimes increased and sometimes decreased with the force level. We believe that the local minimum problem previously mentioned has an impact on the MLE performance in this high-noise scenario, and we did not identify a monotonic relation between the force level and the measured error.

The results in Fig. 4 and Tables 1 and 2 were obtained from simulated S-EMG signals, using 3-s windows—i.e., the entire signal length—, and seven S-EMG-channels. We now evaluate the influence of window length and number of channels on CV estimation.

#### Influence of window length

**Fig. 5:**
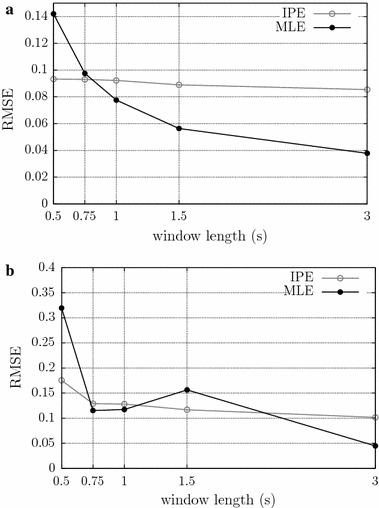
Evaluation of the influence of window lengths on CV estimation in the MLE and IPE methods, for a force level of 20 % of the MVC. The root mean squared error is shown as a function of the length of the window used to estimate the CV, for simulated signals with CV of 4 m/s, at different noise levels: **a** noise free; and **b** 16 dB SNR

Figure 5 shows the results of the experiments which evaluated the influence of window length on CV estimation, considering a force level of 20 % of the MVC. For noise free signals, the result for the MLE shows RMSE reducing slightly as the window length increases, the IPE method has a constant behavior. At 0.75 s, both algorithms have similar behavior. For 16 dB SNR signals, results for the MLE and IPE algorithms were very similar, with the RMSE reducing slightly as the window length increases. Overall, the performance of the MLE method was slightly superior to that of the proposed algorithm, which performed better than MLE only for windows lengths of 0.5 s (Fig. 5a). As expected, the use of long windows is more advantageous when the SNR is low. At high SNR, the window length may be decreased in order to improve the temporal resolution.

Results are shown only for 4 m/s signals, because the results for different CV values were similar in bevahiour. Similarly, results are shown only for noise free and 16 dB SNR signals.

#### Influence of the number of channels

**Fig. 6:**
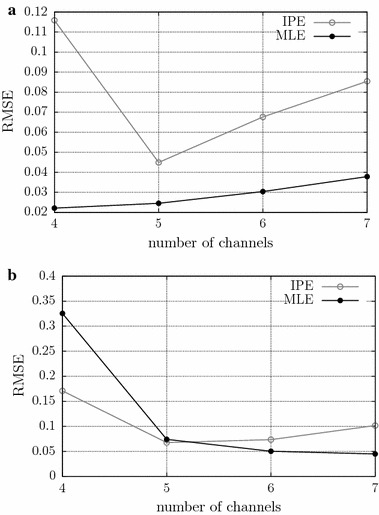
Evaluation of the influence of the number of channels on CV estimation in the MLE and IPE methods, for a force level of 20 % of the MVC. The root mean squared error is shown as a function of the length of the window used to estimate the CV, for simulated signals with CV of 4 m/s, at different noise levels: **a** noise free; and **b** 16 dB SNR

Figure 6 shows the results of the experiments which evaluated the influence of the number of channels on CV estimation, considering a force level of 20 % of the MVC. For 16 dB SNR signals (Fig. 6b), both, MLE and IPE methods have similar behavior, with RMSE decreasing while the number channels used to CV estimation increases. For noise free signals (Fig. 6a), both algorithms presents the RMSE increasing with channel (except for 5 channels in MLE method). In all cases, it is evident that the use of 5 or more channels is recommended to avoid RMSE higher than the high-goodness threshold.

### CV experiments using real biceps brachii S-EMG signals

**Fig. 7:**
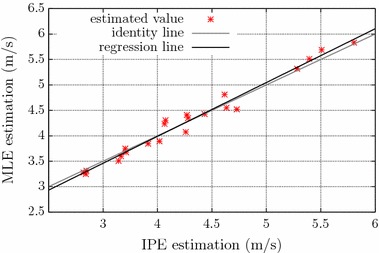
Scatter plot comparing MLE- and IPE-measured CV values from 22 S-EMG signals measured on the biceps brachii muscle of healthy volunteers, during 10-s isometric contractions, at 20 % of the MVC. The *red dashed line* is the identity *line* and the *green dashed line* is the regression *line*. The correlation coefficient was 0.99 ($p < 0.01$), the slope of the regression line was 1.06, and its linear coefficient was −0.24 m/s

Figure 7 shows a scatter plot comparing MLE- and IPE-measured CV values from 22 S-EMG signals measured on the biceps brachii muscle of healthy volunteers, during 10-s isometric contractions, at 20 % of the MVC. Five more signals were evaluated with both algorithm but due to outlier results on the MLE method, this number was reduced to 22 and the outliers were evaluated separately. The green dashed line is the regression line and the red dashed line is the identity line. The results show a strong linear correlation between the two algorithms (correlation coefficient was 0.99, $p < 0.01$), which indicates good agreement. The slope of the regression line was 1.06, and its linear coefficient was −0.24 m/s. If we include the outliers, the correlation coefficient drops to 0.77. In all outlier cases, the MLE method results in non-physiological CV values—6.3, 7.6, 8.0, 8.9 and 9.4 m/s, whereas the IPE method estimated values were respectively 4.5, 4.3, 5.6, 7.0 and 5.0 m/s. These IPE estimates are closer to physiological values, which suggest lower errors, but we don’t know the real mean CV for these signals. In all cases of outliers we noted a high number of MUAP superpositions, which may be the reason for the MLE error. These results suggest that the IPE method is less sensitive to MUAP superpositions than MLE.

## Discussion

The experiments using simulated signals showed that it is possible to robustly estimate the average CV of multichannel S-EMG signals using image processing techniques. When the CV was 3, 4 or 5 m/s, and the SNR was high (≥16 dB), the proposed IPE algorithm was as precise and accurate as the MLE method proposed by Farina et al. [12]. When the SNR was low (=12 dB), the MLE method performed not as well as the IPE method, but generally provided acceptable results (RMSE $<$0.5 m/s, which still allows detecting variations of the average CV [12]). The performance of the IPE algorithm degrades when the SNR is low, because false conduction lines due to noise remain even after morphological transformation. However, IPE algorithm performed better than MLE for these SNR.

It is important to note, however, that signals with SNR lower than 20 dB are not commonly used. Noise levels in adequately-performed S-EMG experiments are typically on the order of 2–10 µVrms, while typical amplitude values for S-EMG signals are on the order of 4–5 mVpp [37].

The fact that IPE method performed better than the MLE method may be because the IPE algorithm does not rely on an initial guess for the CV. The issue of CV initialization can be critical when using the MLE method, especially when the SNR is low. When the true CV is considerably higher than the user-defined initial guess, the MLE method tends to provide incorrect CV estimates. Similarly, if the wrong direction of MUAP propagation is assumed, the MLE algorithm tends to provide highly imprecise estimates. These issues with the MLE algorithm are due to local minima problems associated with the Newton–Raphson method. The proposed IPE method does not use iterative optimization methods, therefore it is not susceptible to this type of error, and does not require any a priori information about the direction of MUAP propagation.

For all the simulated signals we used, the channels were away from the IZs. In preliminary experiments with simulated signals containing one IZ, the proposed method showed better results than the MLE method. However, neither method should be used to estimate the CVs on signals containing IZs, without modification. The MLE algorithm, as originally proposed by Farina et al. [12], estimates the amount of delay between adjacent channels by minimizing the MSE between signals present in these two channels. This delay, however, is positive for the channels positioned before the IZ and negative for the channels positioned after the IZ. The MLE algorithm does not take this into consideration, and therefore is not able to accurately estimate the CV for signals containing IZs. The IPE method is potentially able to accurately estimate the CVs from conducting lines on both sides of the IZ. However, the conduction lines are generally curved near the IZ because of non-propagating potentials. Since the CV is calculated from the slope of the conduction lines, the image’s rows corresponding to channels near the IZs should be discarded before calculating the CV. An automatic IZ tracking process [26, 30], followed by removal of the channels containing IZs, could be incorporated into the IPE algorithm in order to allow its use with signals containing IZs. This idea will be explored in future works.

When applied to biceps brachii signals, the proposed method showed a similar performance of CV with respect to the MLE method. However, the MLE method estimated non-physiological CV values for 5 real signals. For this motive these signals were discarded, however, IPE method was able to estimate physiological values for these cases.

## Conclusion

This work proposed, demonstrated, and evaluated an image-based S-EMG analysis algorithm for estimation of MUAP conduction velocity. We showed that it is possible to robustly estimate the average CV of multichannel S-EMG signals using image processing techniques. The proposed IPE algorithm is as precise and accurate as the MLE method proposed by Farina et al. [12] in typical conditions of SNR and CV. The proposed IPE method does not use iterative optimization methods, therefore it is not susceptible to errors associated with MUAP propagation direction or inadequate initialization parameters, which are common with the MLE algorithm. An automatic IZ tracking process could be incorporated into the IPE algorithm in order to allow its use with signals containing IZs.

Image processing-based approaches may be useful in S-EMG analysis to extract different physiological parameters from multichannel S-EMG signals. A wide variety of new methods based on image analysis could be developed to estimate parameters that are difficult to assess using traditional methods, such as CV. Problems that could be investigated using image processing techniques (in combination with pattern recognition methods) include: estimating the CV for individual MUs, IZ localization and tracking, EMG decomposition, and study of MU recruitment strategies.
